# Supplementary material for: Shared characteristics underpinning C4 leaf maturation derived from analysis of multiple C3 and C4 species of Flaveria
Source: J Exp Bot. 2017 Jan 6;68(2):177–89. doi: 10.1093/jxb/erw488 (PMC5853325; doi:10.1093/jxb/erw488)
Supplement: Supplementary_Figures_S1_S8 [file erw488_suppl_supplementary_figures_s1_s8.pdf]

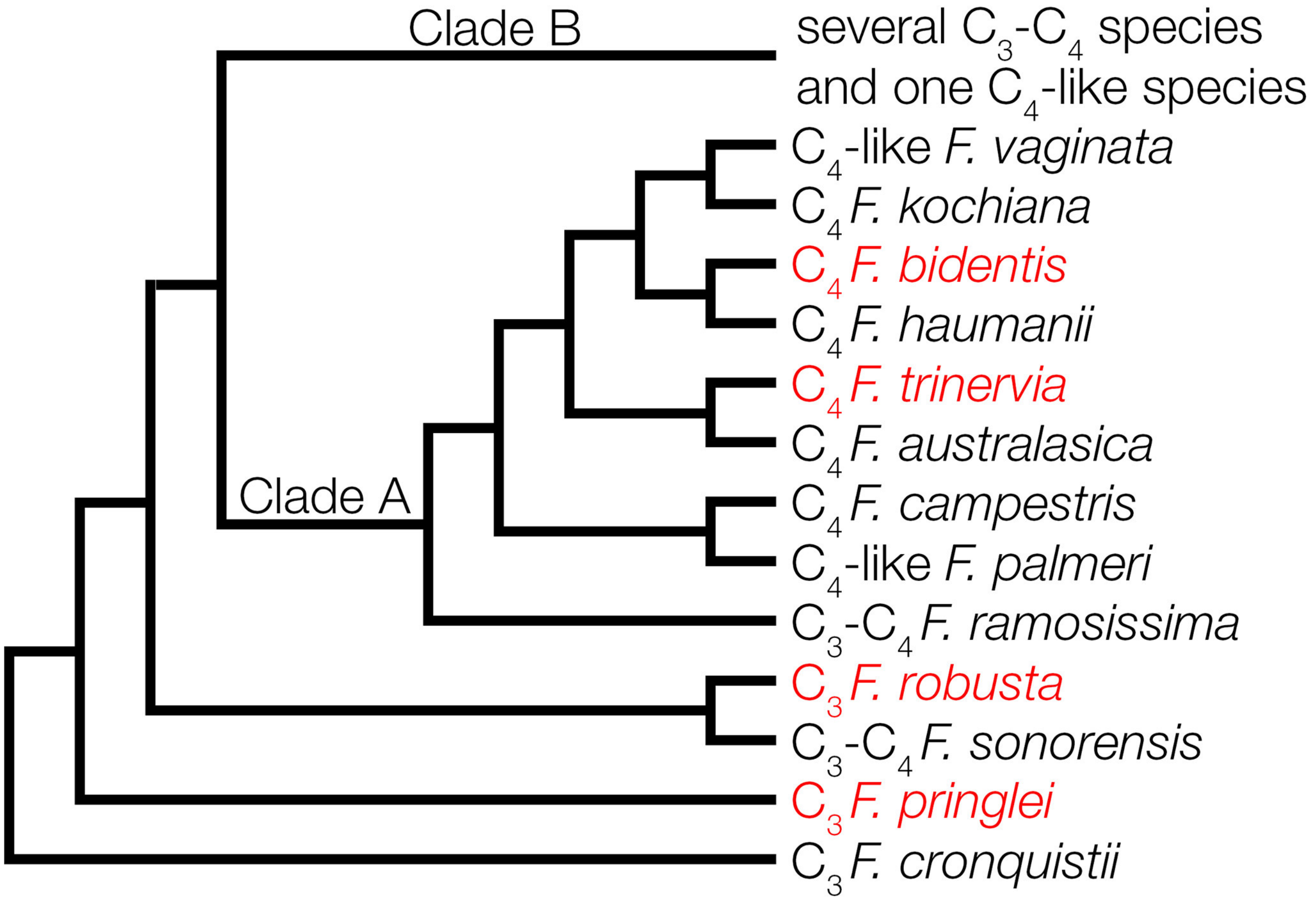

Supplementary Figure 1

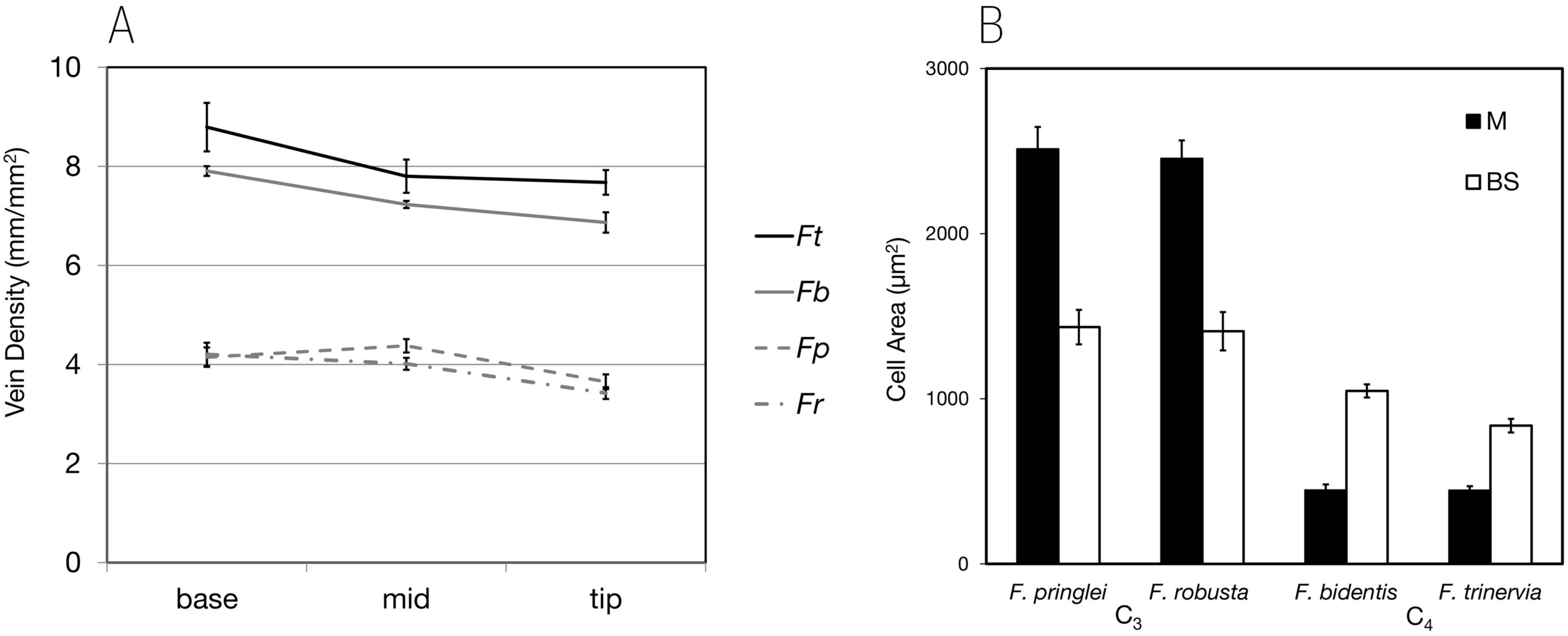

Supplementary Figure 2

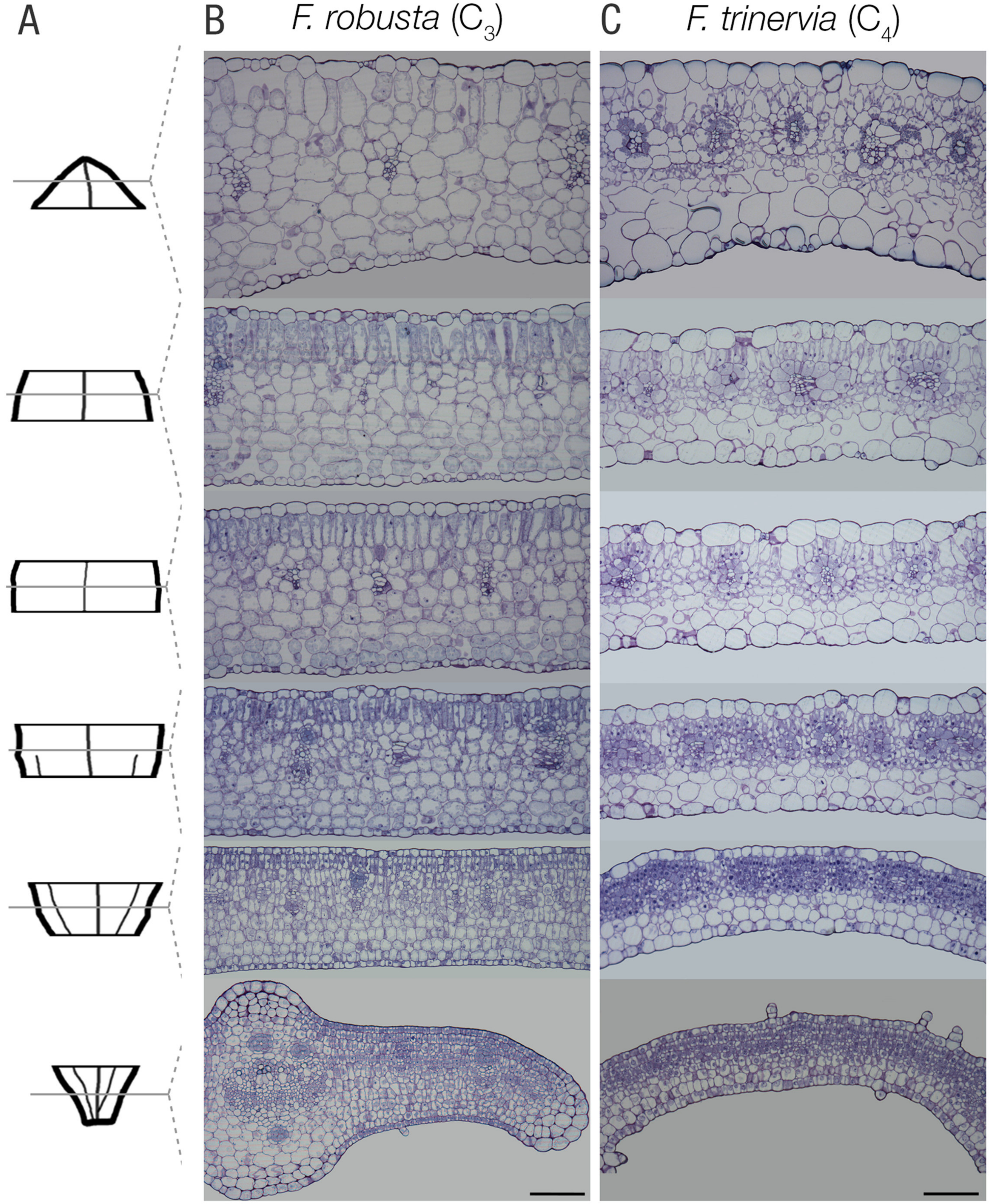

Supplementary Figure 3

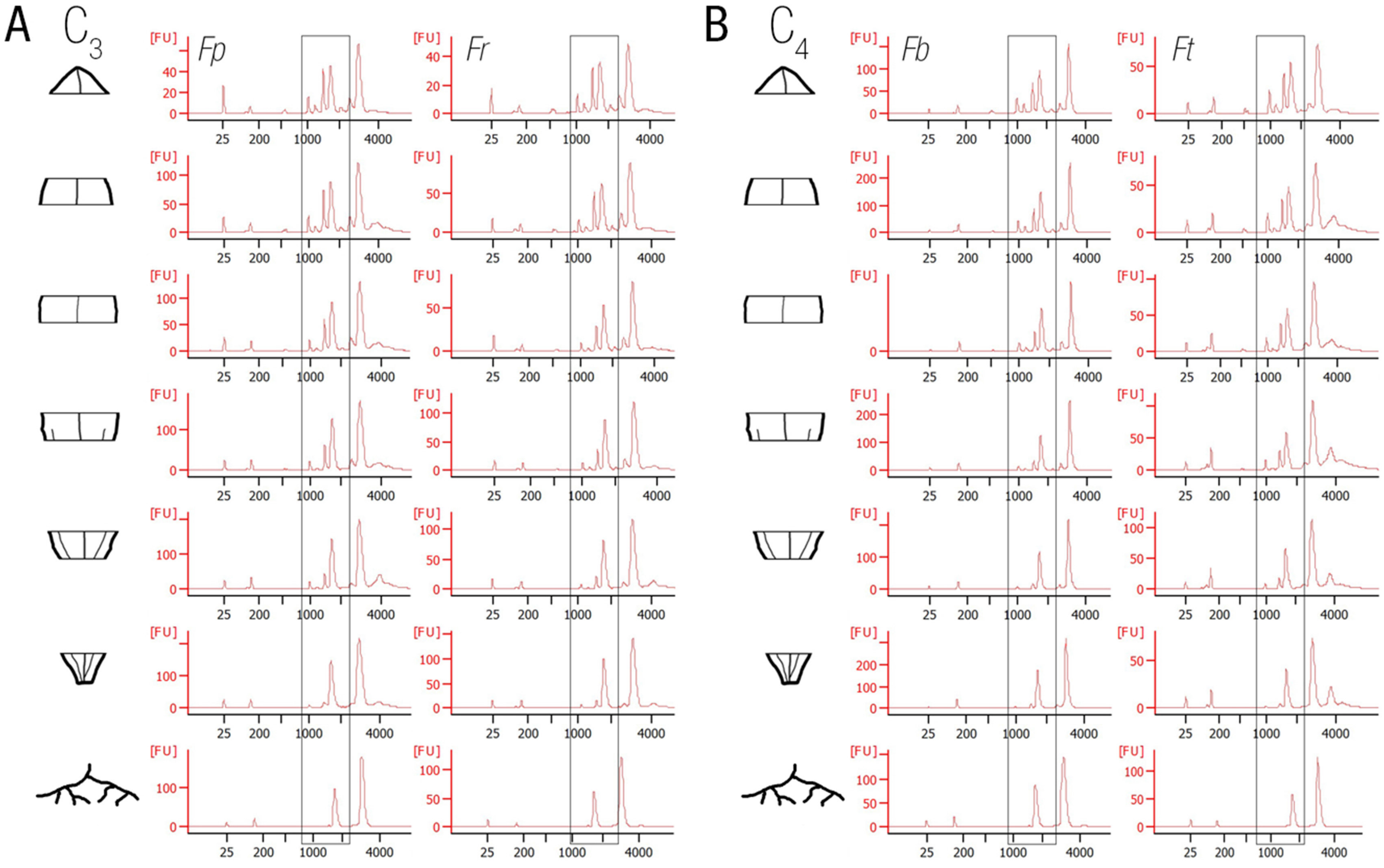

Supplementary Figure 4

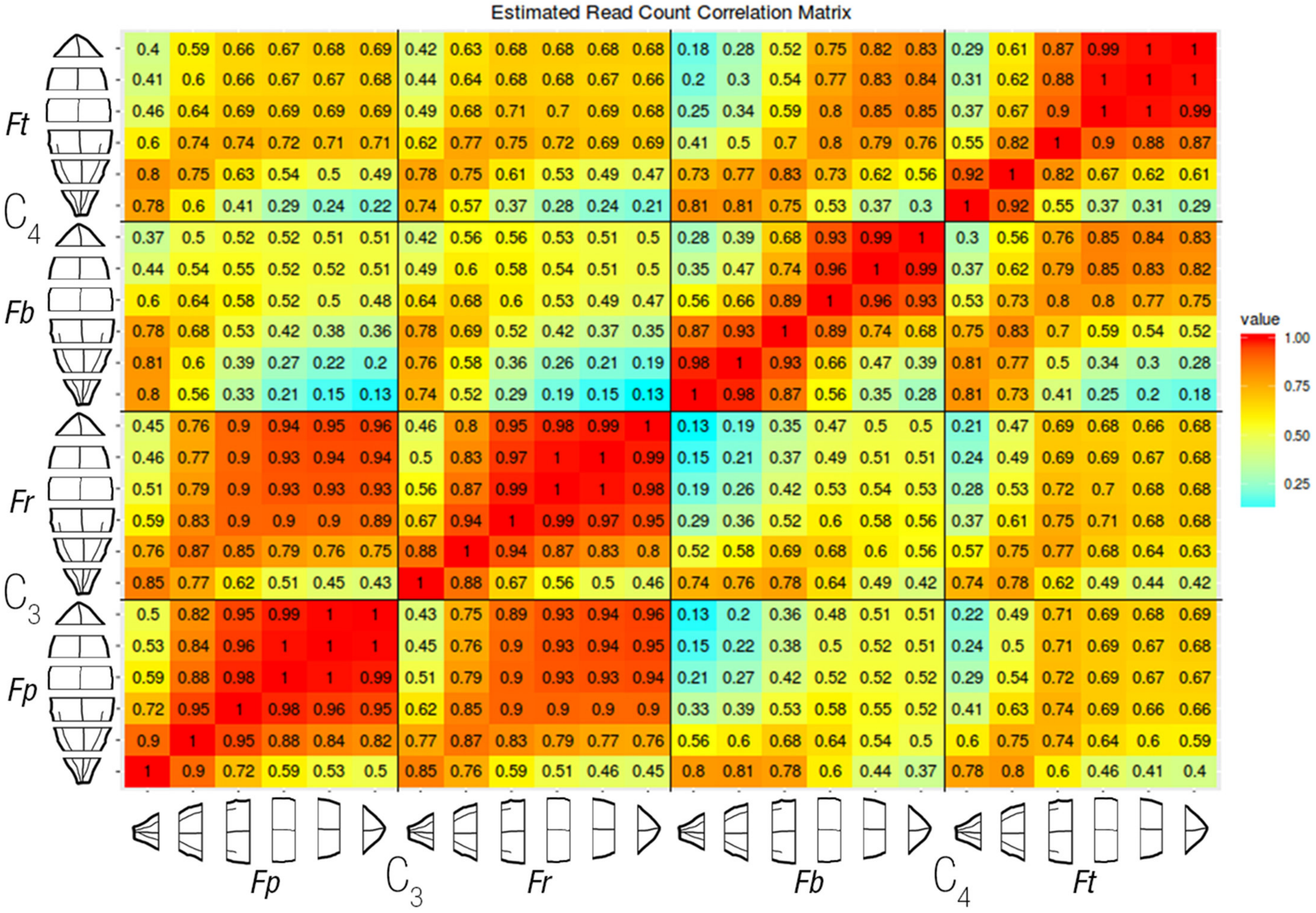

Supplementary Figure 5

Process	*F. pringlei* (C_3)						*F. bidentis* (C_4)					
Transcription (DNA templated)	6	7	6	6	6	6	8	8	8	8	8	7
Regulation of Transcription	6	7	6	6	5	6	8	8	8	8	7	7
Translation	51	51	53	51	48	45	43	45	47	45	42	40
Transport	15	15	15	15	15	15	15	15	15	14	14	14
Cell Proliferation	15	11	6	4	4	4	19	17	10	4	4	3
Chloroplast Organisation	30	42	47	47	46	45	16	27	39	45	48	45
Plant Cell Wall Loosening	0	0	0	6	6	6	18	6	12	12	6	6
Plant-type Secondary Cell Wall Biogenesis	5	5	5	5	5	3	12	7	5	5	2	2
Post Transcriptional Regulation of Gene Expression	17	17	16	15	14	14	16	17	15	14	12	11
Photosynthesis	37	45	51	54	55	56	24	37	46	54	58	58
Cell Growth	18	16	14	14	13	13	18	16	14	13	13	13
Secondary Metabolic Process	22	26	28	29	30	29	22	21	24	25	26	28
Signalling	8	8	7	6	7	7	9	8	8	7	7	7
Regulation of DNA Endoreduplication	10	10	5	5	5	5	6	6	0	0	0	0
Ribosome Biogenesis	51	58	59	58	55	53	36	44	50	51	49	46
Primary Metabolic Process	15	15	15	15	15	14	14	14	15	14	14	14
Root Development	15	14	14	12	12	12	19	17	15	13	12	12

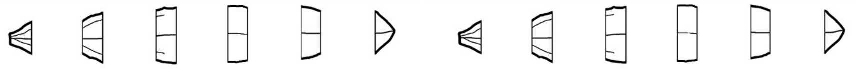

Process	*F. robusta* (C_3)						*F. trinervia* (C_4)					
Transcription (DNA templated)	7	7	7	7	7	7	7	7	8	8	8	8
Regulation of Transcription	7	7	7	7	6	6	7	7	7	7	7	7
Translation	48	50	51	50	48	45	45	50	48	46	42	40
Transport	15	14	15	15	14	15	15	14	15	15	15	15
Cell Proliferation	13	10	5	4	4	3	19	15	7	5	4	4
Chloroplast Organisation	26	42	48	47	45	42	19	35	38	38	39	41
Plant Cell Wall Loosening	5	0	0	0	0	5	38	15	8	8	8	8
Plant-type Secondary Cell Wall Biogenesis	11	5	5	8	5	3	10	8	8	8	5	5
Post Transcriptional Regulation of Gene Expression	17	16	15	14	13	13	16	15	12	12	11	11
Photosynthesis	34	43	48	50	51	50	33	45	52	56	58	60
Cell Growth	17	14	12	12	12	12	20	16	14	13	13	13
Secondary Metabolic Process	27	27	28	30	30	30	23	23	26	28	29	29
Signalling	9	8	8	7	7	7	9	8	8	8	8	9
Regulation of DNA Endoreduplication	17	11	6	6	6	6	11	11	6	0	0	0
Ribosome Biogenesis	44	53	56	55	52	49	42	53	53	50	49	47
Primary Metabolic Process	15	15	15	15	14	14	14	14	14	14	14	14
Root Development	16	13	12	12	12	10	16	13	12	12	11	11

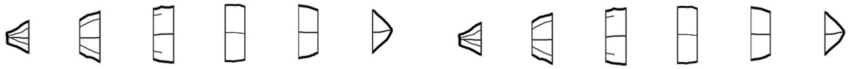

Supplementary Figure 6

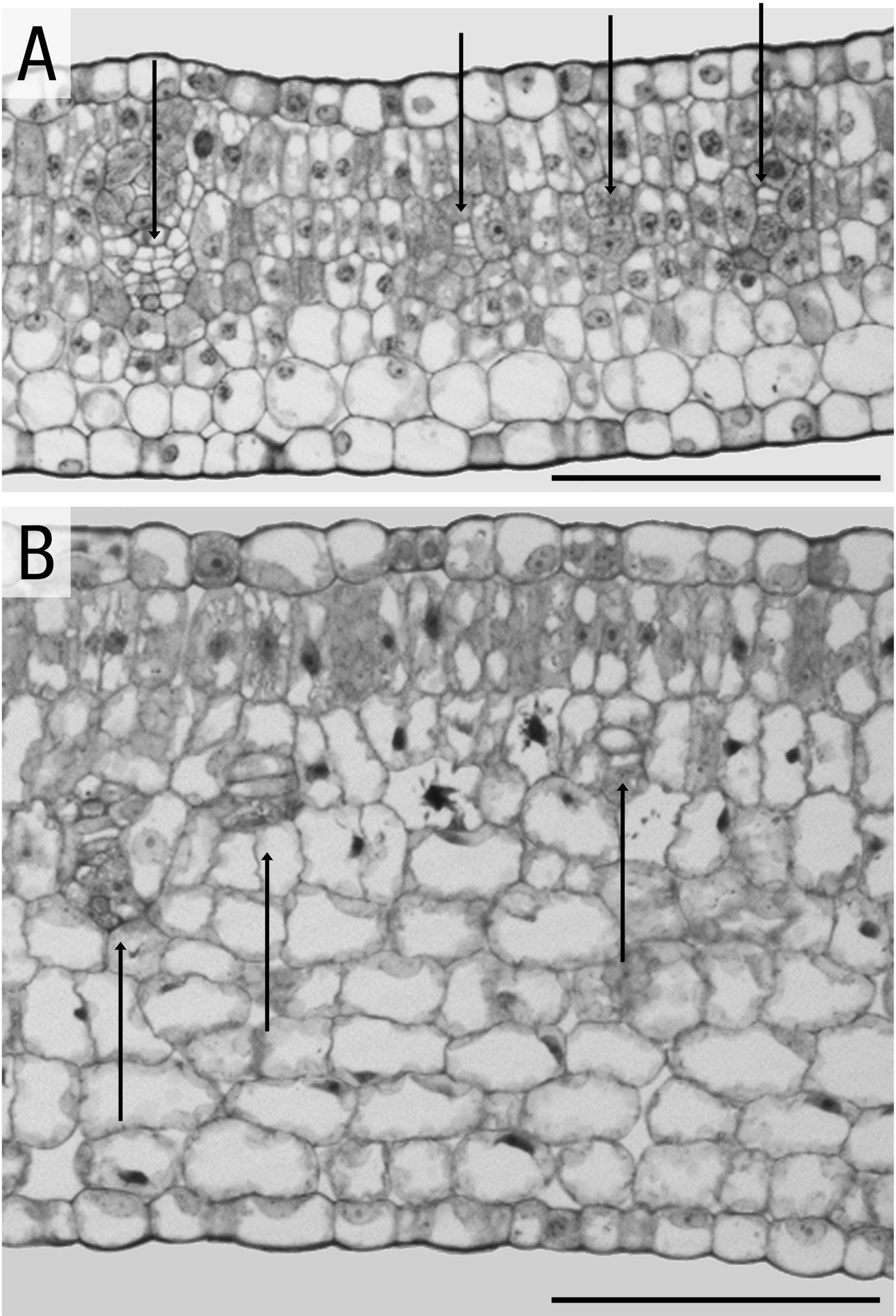

Supplementary Figure 7

Supplementary Figure 8.A

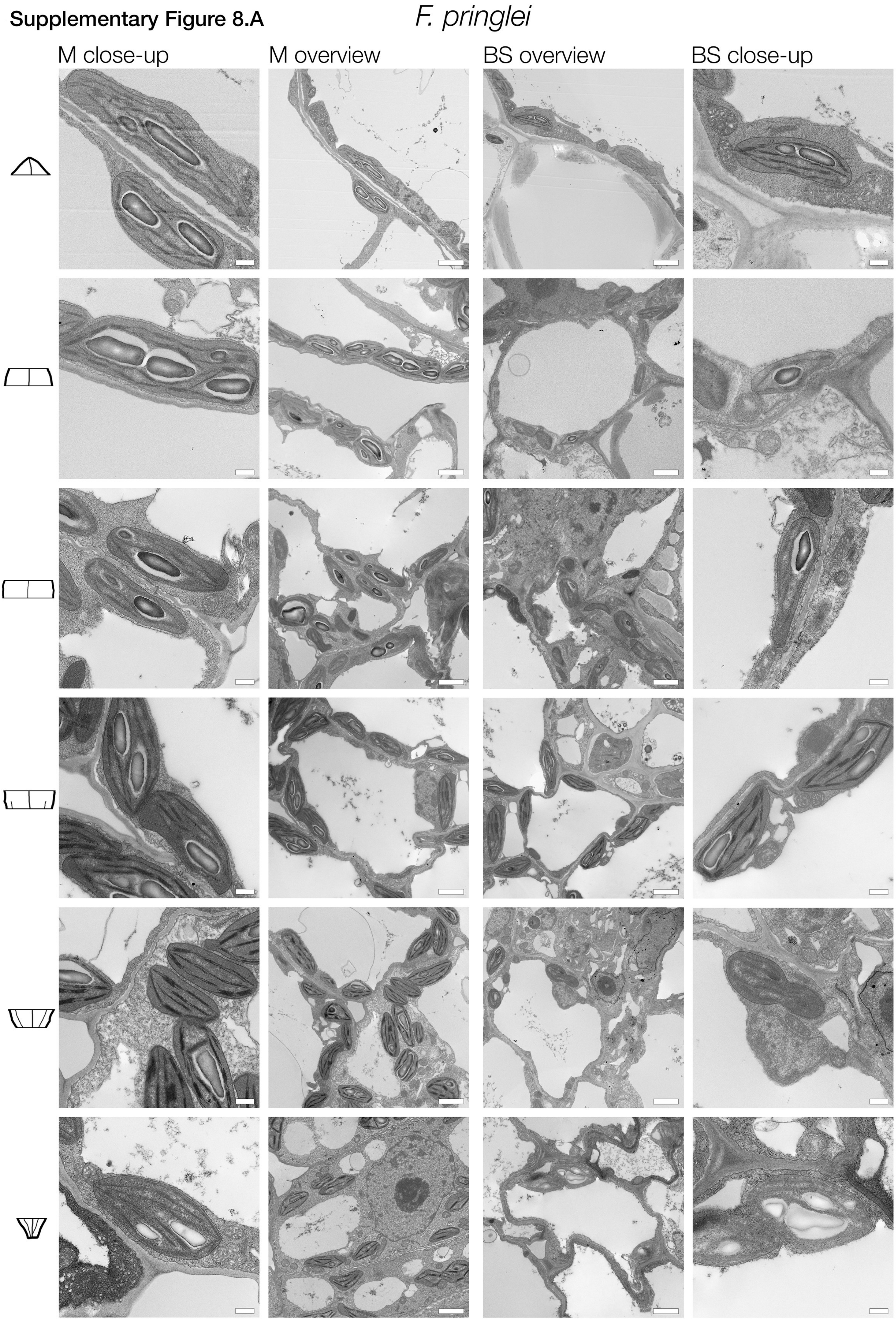

Supplementary Figure 8.B

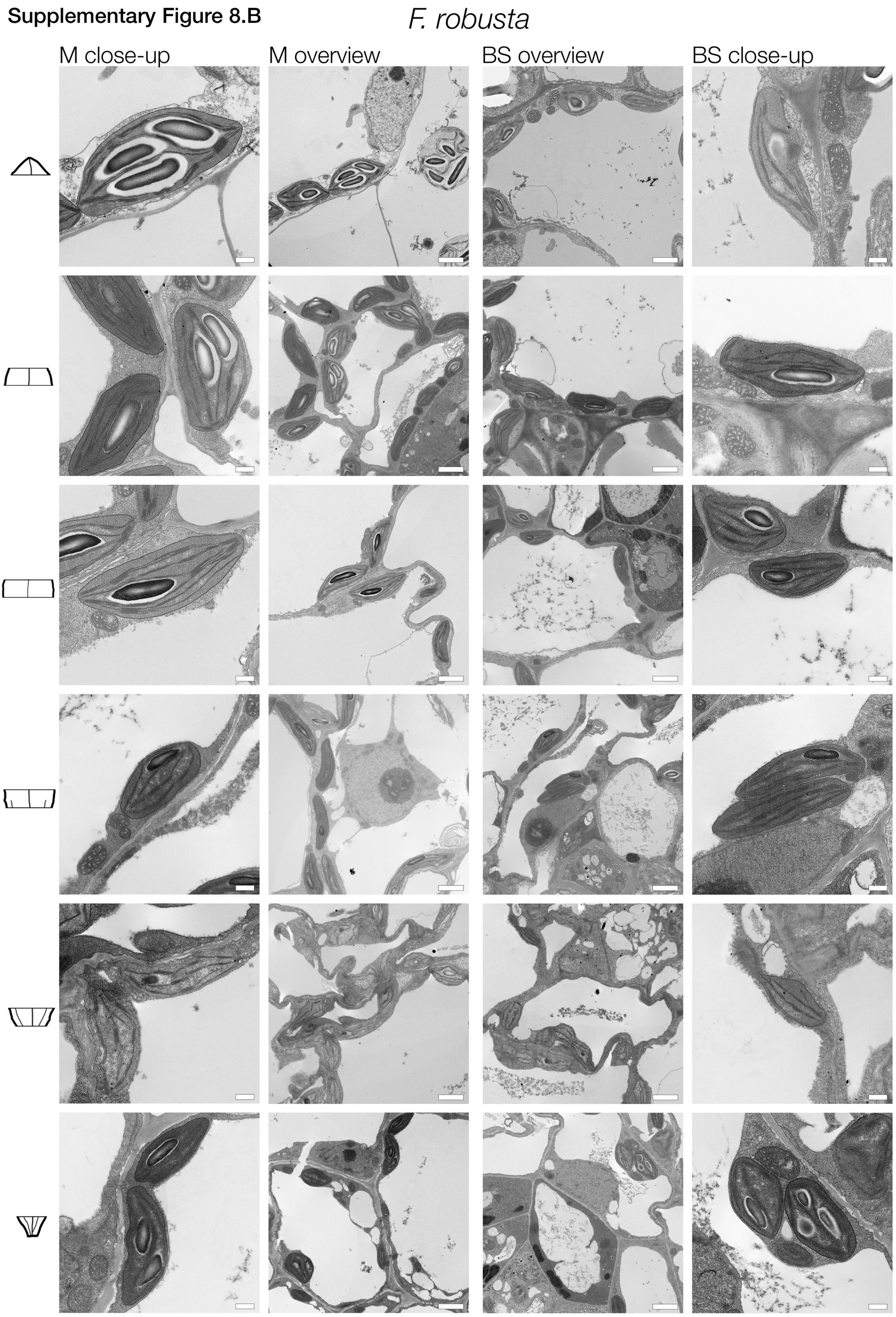

Supplementary Figure 8.C

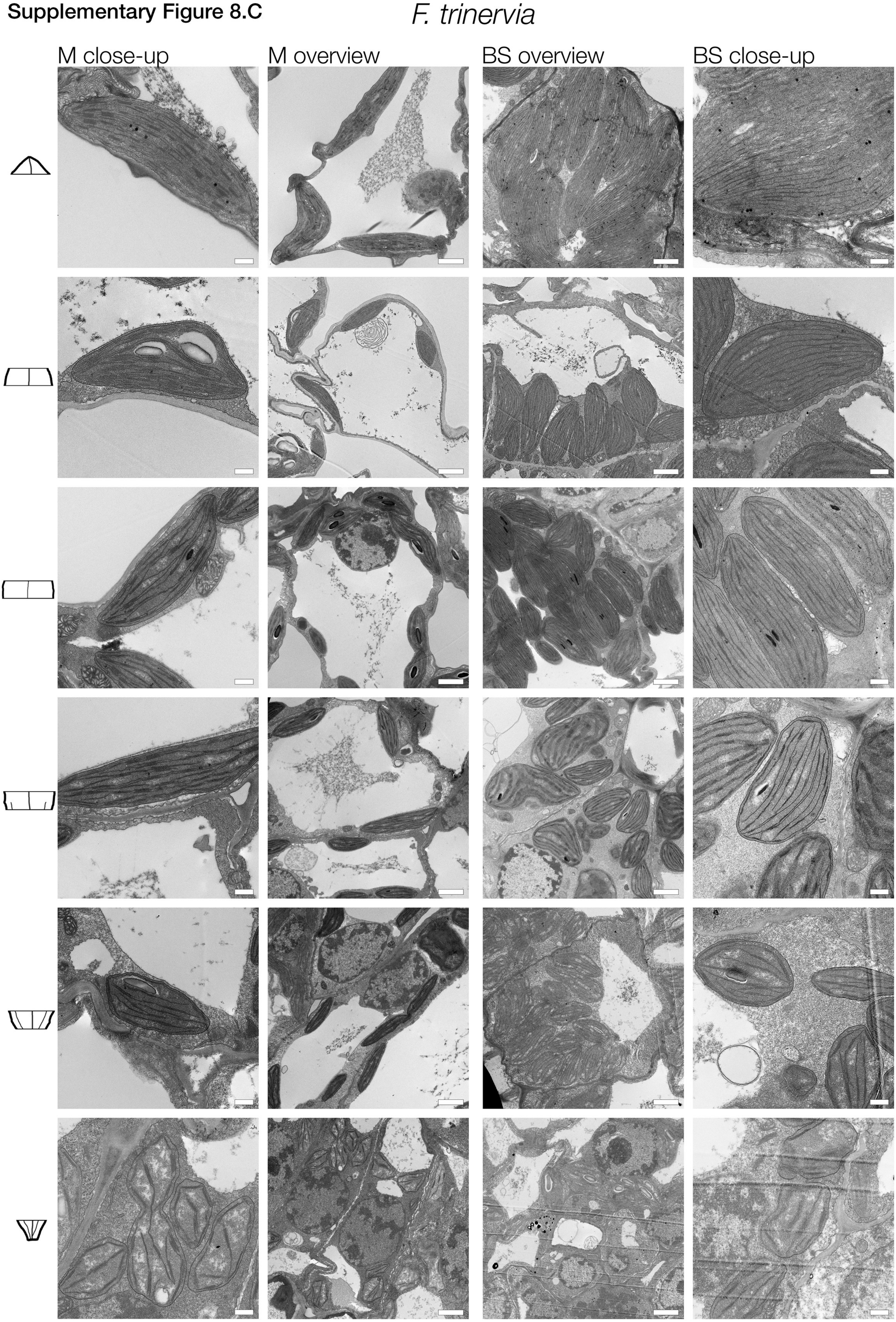

Supplementary Figure 8.D

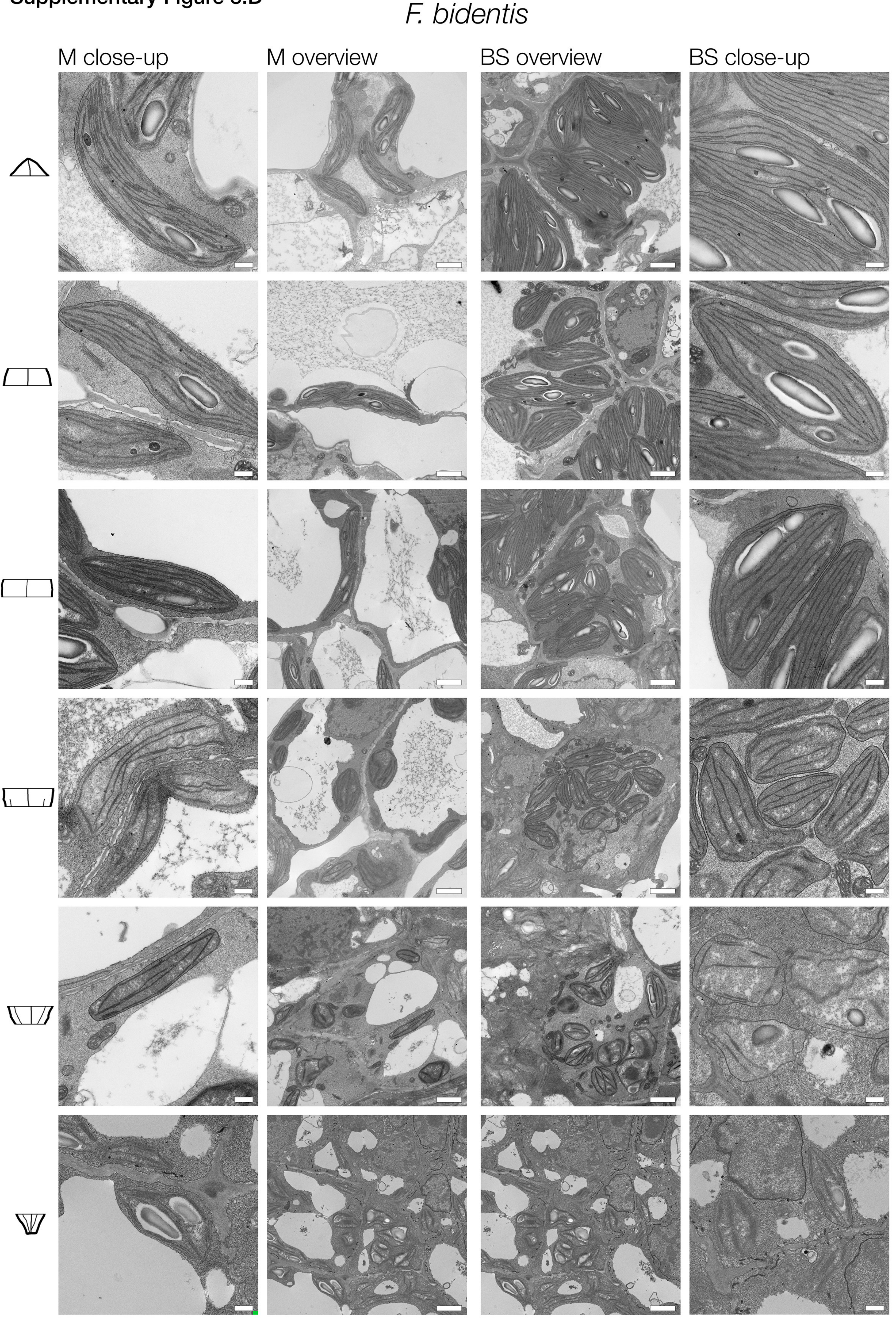

Supplementary Figure legends

Supplementary Figure 1: *Flaveria* phylogeny adapted from McKown and Dengler 2007. This phylogeny was used as it contains all the species analysed in this study (marked in red).

Supplementary Figure 2: Mature leaf vein density and cell size**. A**: Vein density at the base, mid and tip of mature leaves of the four *Flaveria* species analysed. **B**: Cross-sectional area of mesophyll (M) and bundle sheath (BS) cells in the middle of mature *Flaveria* leaves.

Supplementary Figure 3: Leaf anatomy maturation gradient in C_3 and C_4 *Flaveria*. **A**: Representative leaf outline illustrating leaf sampling. Representative transverse sections from C_3 *F. robusta* (**B**) and C_4 *F. trinervia* (**C**) from base (bottom) to tip (top). Note the gradual expansion of cells, increased vacuolisation and clearer delineation of both mesophyll and bundle sheath cells from base to tip. Scale bars represent 100µm.

Supplementary Figure 4: RNA quality from sections of each species. **A&B**: Representative RNA electropherograms from C_3 *F. pringlei* and *F. robusta* (**A**) and C_4 *F. bidentis* and *F. trinervia* (**B**) leaf samples from base to tip. Root RNA is provided as control. Note the gradual increase in chloroplast RNA peaks (not detectable in root samples) in the electropherograms as the leaves mature. Y-axes show arbitrary fluorescence units and X-axis the RNA length in nucleotides.

Supplementary Figure 5: TPM correlation matrix. Pearson's correlation analysis was undertaken on TPM values at each sampling point using the 8000 annotations found in all four species. Leaf outlines indicate which part of the leaf was sampled. *Ft* = *Flaveria trinervia*, *Fb* = *Flaveria bidentis*, *Fr* = *Flaveria robusta*, *Fp* = *Flaveria pringlei*.

Supplementary Figure 6: GO term analysis presented as heatmaps for each *Flaveria* species showing the percentage of genes from an individual GO category found in the top 10 percentile of all detected genes for that species. Leaf outlines indicate sampling stage. For example a 40 at the leaf tip in the GO category translation, means 40% of the genes involved in translation are in the top 10 % of the highest expressed genes at the leaf tip.

Supplementary Figure 7: Cell division and vein development in the base samples of *Flaveria*. **A**: C_4 *F. bidentis* upper base section shows a developed major vein on the left and three developing veins to the right, with the vein (2nd from the right) showing the first cell division that sub-divides one cell into three. The middle cell then goes on to divide again and the top and bottom likely become bundle sheath cells. The vein to the right shows one more division of the middle cell which will form the vein and the surrounding cells starting to change shape towards the typically bundle sheath shape. The vein second from the left has yet more divisions of the cell forming the vein **B**: C_3 *F. pringlei* upper base section and 2 developing veins to the right. All veins are marked with arrows. Scale bars represent 100µm.

Supplementary Figure 8: Chloroplast maturation in mesophyll and bundle sheath cells for each species. Chloroplast development in BS and M cells from base to tip in *Flaveria pringlei* (A)*, Flaveria robusta* (B)*, Flaveria trinervia* (C) and *Flaveria bidentis* (D). Scale bars represent 500nm in close-up images and 2µm in overview images.
